# Microwave-sensor-node integrated into a short-range wireless sensor network

**DOI:** 10.1038/s41598-023-28964-8

**Published:** 2023-02-06

**Authors:** Miguel Hernandez-Aguila, Jose-Luis Olvera-Cervantes, Aldo-Eleazar Perez-Ramos, Juan-Mateo Meza-Arenas, Alonso Corona-Chavez

**Affiliations:** 1 Instituto Nacional de Astrofísica, Óptica Y Electrónica, Puebla, Mexico; 2 Departamento de Ingeniería Electrónica, TecNM – Instituto Tecnológico de Oaxaca, Oaxaca, Mexico

**Keywords:** Engineering, Electrical and electronic engineering

## Abstract

This paper presents the first microwave-sensor-node integrated into a short-range wireless sensor network based on ZigBee technology. The node includes an analog front-end circuit, a Frequency Modulated Continuous Wave generator, an Analog-to-Digital-Converter module, a transceiver, a power unit, a processing unit and a new one-port dielectric permittivity sensor which is able to measuring the separation of structural cracks by the reflection coefficient measured in microwave frequencies. The analog front-end is composed of a pair of power dividers, an isolator and a mixer. The dielectric permittivity sensor is based on a patch antenna of variable length. The processing unit and transceiver are implemented with an Arduino UNO and an XBee module respectively. Additionally, the methodology for data processing is presented and the results of the measurement of a synthetic crack are presented. The results show that the system was successfully implemented with a sensitivity of 0.07 GHz/mm, for an opening range of between 0 and 5 mm and for a frequency range ranging from 2.782 GHz to 3.131 GHz. It is important to mention that the measurement was done remotely, placing the sensor 3 m from the client PC.

## Introduction

Wireless Sensor Network (WSN) is defined as a set of sensor nodes that sense data in one medium and send it to a user, in addition, based on the sensor used the characteristics of the sensor nodes can differ between one and another. WSNs are currently a growing technology trend because they have a low installation cost, high mobility capacity and do not require cabling. So WSNs can offer advantages in areas such as agriculture, industry, the military, health care, the environment, among others^1^.

**Figure 1:**
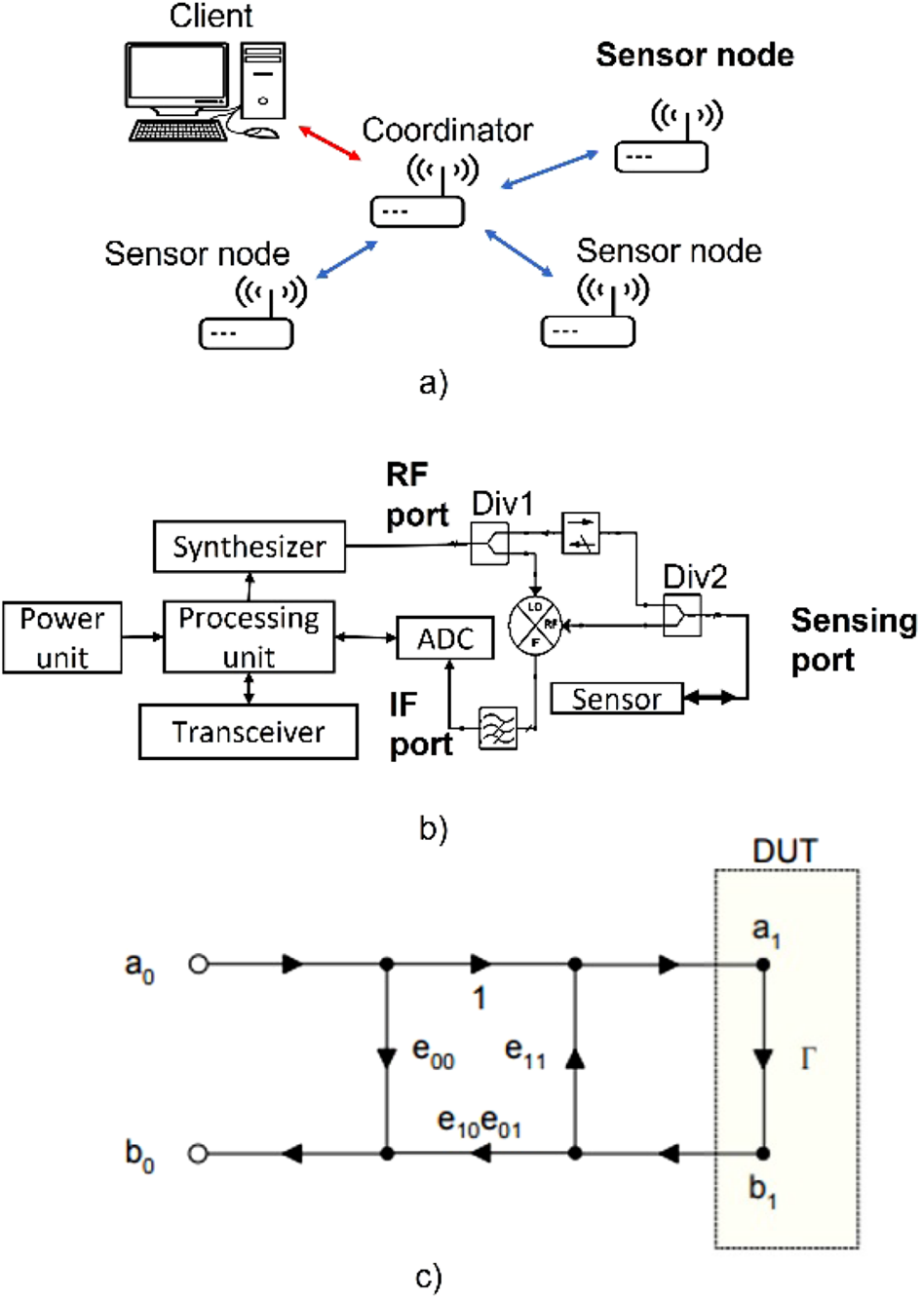
Diagram of (**a**) a ZigBee WSN, (**b**) Analog front-end and (**c**) Three-term error model^37^.

WSNs can be classified as short-range, long-range and heterogeneous WSN. Short-range WSN are usually implemented through different communication protocols such as ZigBee, Bluetooth LE, RFID, WLAN, Z-Wave, Thread, etc^2,3^. ZigBee-based short-range networks are widely used because it has a maximum data transfer rate of 151 Kb/s, can reach a range of up to 20 m, allows working with networks of up to 64,000 sensor nodes and supports mesh, ad hoc and star topologies^3,4^. ZigBee networks are made up of a client, a coordinator and an end- device that is connected to a sensor node as shown in Fig. 1. The client provides the user with an interface for the control of the network while the coordinator is responsible for communicating the client’s orders and collecting the data from the sensor nodes.

A sensor node consists of a power unit, a processing unit, a transceiver, and a sensing unit^2^. The power unit energizes the entire sensor-node, the processing unit controls all operations that are carried out by the sensor node and the transceiver allows the sensor node to establish communication with the WSN. The sensing unit is the part of the sensor node responsible for capturing physical quantities such as humidity^5^, gases^6^, ph^7^, temperature^8^, sound^9^, dielectric permittivity^10–13^, etc.

Dielectric permittivity sensors are a type of sensors that relate dielectric permittivity in microwave frequencies to physical variables. Many dielectric permittivity sensors have been proposed worldwide for applications in non-invasive glucose detection^14^, milk quality monitoring^15^, humidity measurement^16^, liquid characterization^17^, chromatography^18^, meat quality^19^, food adulteration^20^, temperature measurement^21^, pH measurement^22^, gas detection^23^, diabetic foot ulcer detection^24^, breast cancer detection^25^, ultraviolet light detection^26^, salt and sugar measurement in water^27^, characterization of electrolytes in urine^28^, moisture in tissues measurement^29^, chemical reactions in gases^30^, measurement of small displacements^31–35^, etc.

Of the works mentioned above, only the sensor developed in Jilani^29^ has been connected to a short-range WSN by using a vector network analyzer (VNA) equipped with a Bluetooth module. The scheme proposed by Jilani^29^ has disadvantages associated with the use of a VNA; the two main disadvantages are the high cost of the system and the inability to implement networks with multiple dielectric permittivity sensors, since one VNA per sensor is required.

In this work, a new displacement sensor is proposed for the measurement of high-precision structural cracks, which is measured through the standard deviation. The sensor operates from 2.5 to 3.1 GHz with a sensitivity of 0.07 GHz/mm and a resolution of 0.5 mm. The sensor was developed with a single port and integrated front-end circuitry to introduce the first microwave sensor-node that has been successfully connected to a short-range wireless network based on ZeegBee technology. The main advantage of the proposed system is that the sensor-node has been integrated into a wireless network without the need for a vector network analyzer, therefore the proposed system is a low-cost system suitable for the implementation of dielectric permittivity sensor networks with multiple sensors. The sensor node uses a displacement sensor based on a patch antenna and an analog front-end circuit to perform the sensor reading. The analog front-end circuit consists of a synthesizer, an isolator, two Wilkinson-type power dividers and a mixer. The output signal of the front-end circuit is digitized with the ADC of an Arduino UNO. The processing unit and transceiver are implemented through an Arduino UNO and an XBee module respectively. On the other hand, the network consists of a client PC made through a PC with Matlab 2018 connected to the sensor node through a Coordinator device implemented through an XBee module and a TTL-USB converter. This work includes a methodology for signal processing and front-end calibration as well as the methodology for wireless communication between the sensor node and the client PC. Finally, a comparison of our work with other previously reported works is included.

## Proposed sensor node and its connection to a ZigBee network

The diagram of the proposed sensor node is shown in Fig. 1b. The node includes an analog front-end circuit, a Frequency Modulated Continuous Wave (FMCW) generator, an ADC module, a transceiver, a power unit, a processing unit, and a dielectric permittivity sensor. The analog front-end is composed of a pair of power dividers, an isolator and a mixer.

The principle of operation of the front-end circuit is as follows: a chirp signal enters the RF port of the analog front-end and is directed towards the power splitter Div1 where the signal is divided into two signals from which one is connected on the mixer’s LO port and the other is guided to the microwave sensor through the sensing port. In the sensing port there is a wave emerging towards the sensor and a wave reflected from the sensor; the reflected signal is divided into two branches within the divider Div2 from which one is removed with the isolator and the other is guided towards the mixer’s RF port. The RF and LO signal in the mixer are combined to have a few Hertz frequency signal (IF signal), which is filtered and digitized by the ADC.

### Microwave sensor

**Figure 2:**
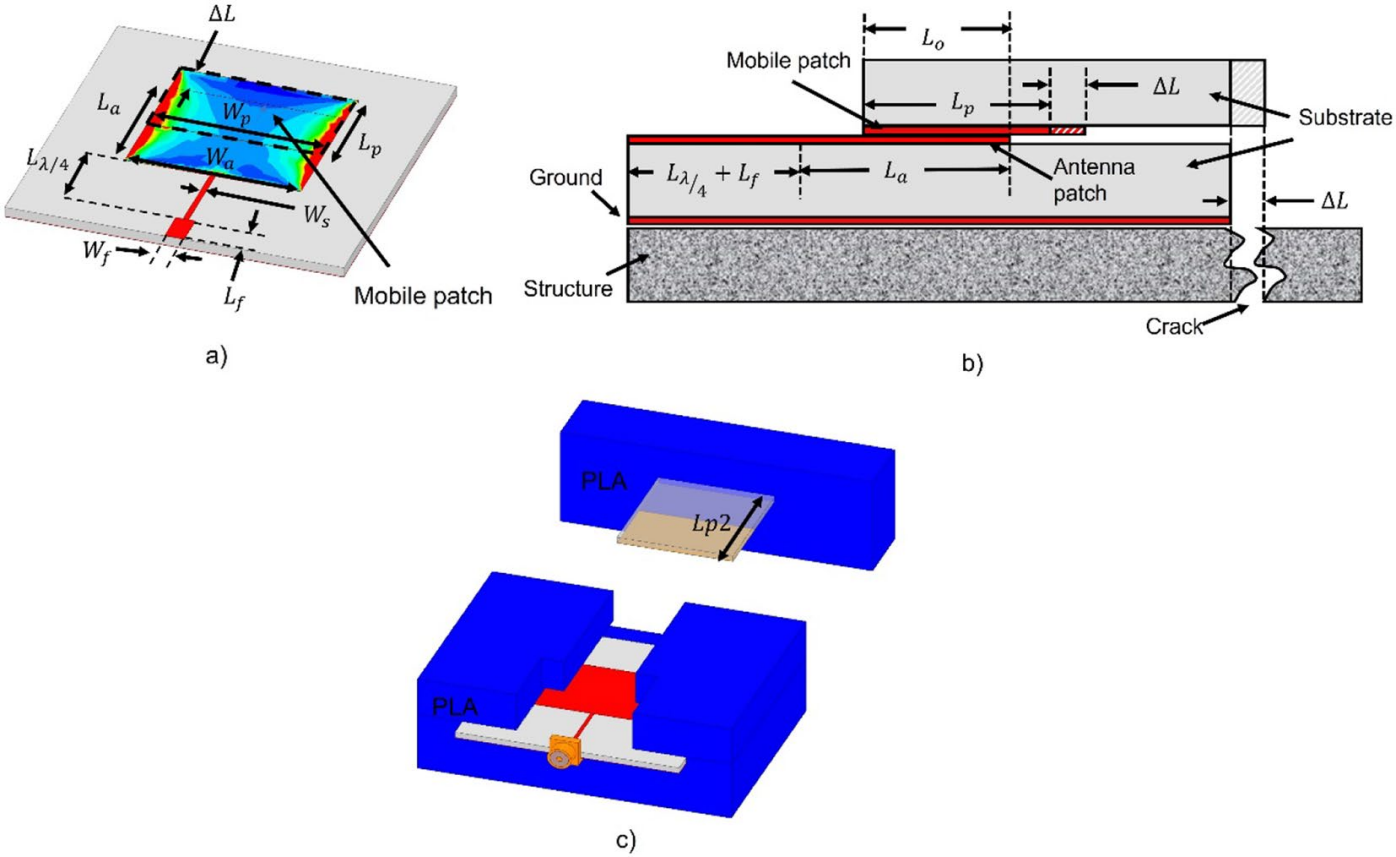
(**a**) Patch antenna together with overlaid mobile patch, (**b**) Longitudinal cut of the proposed sensor mounted on a cracked structure and (**c**) Proposed 3D sensor model with insulation housing made of 3D printer.

The sensor is based on a rectangular patch antenna of width $W_{a}$ and length $L_{a}$, a quarter-wave impedance transformer of width $W_{s}$ and length $L_{\lambda /4}$ and a mobile patch of width $W_{p}$ and length $L_{p}$ which overlaps on the antenna as shown in Fig. 2a. A SMA connector is attached on the 50 Ω microstrip transmission line whose dimensions are $W_{f}$ and $L_{f}$.

The mechanism of operation of the proposed sensor is based on Eq. (1) which represents the resonance frequency $f_{r10}$ of the dominant mode of the antenna considered as a rectangular cavity whose length $L_{a}$, width $W_{a}$ and height $h$ respect that $L_{a} > W_{a} > > h$ ^36^. Equation (1) shows that the resonance frequency depends linearly on the antenna length, the thickness ($h$) of the antenna substrate, the effective permittivity ($\epsilon_{e}$) of the substrate, and the speed of light ($c$) in the vacuum. Equation (1) is important because the combination of an antenna and a mobile patch allows the antenna length to be modified artificially and therefore its resonance frequency.

1 $$f_{r10} = \frac{c}{{2L_{a} \sqrt {\epsilon_{e} } }}$$

Figure 2b shows the longitudinal cut of the proposed sensor mounted on a cracked structure; where the antenna and the mobile patch are attached to the edges of the crack whose width is represented by $\Delta L \ge 0$. Under this configuration the resonance frequency of the sensor is given by Eq. (2) where $L_{o}$ is the length of the part of the mobile patch that is in contact with the antenna patch when the sensor is at an initial point. Looking at Fig. 2b it is clear that the maximum measuring range of the sensor is set by $L_{o}$ as it must be respected that $0 \le \Delta L \le L_{o}$.

2 $$\left( {f_{r} } \right)_{10} = \frac{c}{{2[L_{a} + L_{p} - (L_{o} - \Delta L)]\sqrt {\epsilon_{e} } }}$$

The sensor in Fig. 2 is like the proposed by Xue^31^ because they use the concept of overlapping subpatch, however there are significant differences that must be mentioned. The sensor in Xue^31^ has no power ports while the sensor in Fig. 2 is a one-port sensor coupled by a quarter lambda transformer. The presence or absence of a port coupled in the sensors also causes significant differences in the way the resonance frequency is measured and in the possibility of integrating them into wireless networks. To measure the resonance frequency Xue^31^ uses a VNA and a broadband antenna separated 20 mm from the sensor; in this sense to integrate the sensor into a network requires connecting the VNA to the network which is not feasible in a network of sensors. Additionally, the system in Xue^31^ is sensitive to parasitic effects such as the interference of the test table and the crack simulator and the variation of the distance between the measuring antenna and the crack sensor. The parasitic effects mentioned above are irrelevant to the system proposed in this work; additionally, the measuring distance with the proposed system is 3 m when the sensor node is integrated into a short-range wireless sensor network based on ZigBee technology.

### Determination of the reflection coefficient using the analog front-end

The signal feeding the analog front-end is a chirp train where each chirp signal $u_{LO} \left( t \right)$ is represented by Eq. (3), where $k = \frac{f}{T} = \frac{{f_{H} - f_{L} }}{T}$, $f_{L}$ is the initial frequency of the sweep, $f_{H}$ is the final frequency, $T$ is the period and $a\left( t \right)$ is the amplitude of the chirp signal. The $u_{LO} \left( t \right)$ signal is guided to the sensor where the $u_{RF} \left( t \right)$ signal is reflected which is given by Eq. (4), where $\Gamma$ is the reflection coefficient at the sensor input and $\tau$ is the delay time between $u_{LO} \left( t \right)$ and $u_{RF} \left( t \right)$. By multiplying Eq. (3) by Eq. (4), complying with $0 \le t \le T$ and $\tau \ll T$ and after filtering the high frequencies an IF signal given by Eq. (5) is obtained at the front-end output where $f_{IF} = k\tau$ and $\left| {\Gamma \left( t \right)} \right|$ and $\phi_{\Gamma } \left( t \right)$ are the magnitude and phase of the sensor reflection coefficient respectively.

3 $$u_{LO} \left( t \right) = a\left( t \right)\cos \left\{ {2\pi \left( {f_{L} t + \frac{k}{2}t^{2} } \right)} \right\}$$

4 $$u_{RF} \left( t \right) = \left| {\Gamma \left( {t - \tau } \right)} \right| \cdot a\left( t \right)\cos \left\{ {2\pi \left( {f_{L} \left( {t - \tau } \right) + \frac{k}{2}\left( {t - \tau } \right)^{2} } \right) + \phi_{\Gamma } \left( t \right)} \right\}$$

5 $$u_{IF} \left( t \right) = \left| {\Gamma \left( t \right)} \right| \cdot \frac{{\left[ {a\left( t \right)} \right]^{2} }}{2} \cdot \Re \left\{ {e^{{j\left\{ {2\pi \left( {f_{L } \tau + f_{IF} t} \right) - \phi_{\Gamma } \left( t \right)} \right\}}} } \right\}$$

To find the reflection coefficient of the sensor, the three-term error model shown in Fig. 1c is adopted (Figure obtained from Rytting^37^). The error model considers errors related to directivity, port coupling and tracking using the terms $e_{00}$, $e_{11}$ and $e_{10} e_{01}$ respectively^37^. Comparing the description of the time-dependent signal with the error model as suggested in Hauschild^38^, it is obtained that the coefficient of reflection of the sensor is given by Eq. (6), where ${\varvec{u}}_{{{\varvec{IF}}}}$ is the analytical signal obtained from the real signal given in Eq. (5) and its Hilbert transform.

6 $${\Gamma } = \frac{{{\varvec{u}}_{{{\varvec{IF}}}} - e_{00} }}{{e_{10} e_{01} + e_{11} \left( {{\varvec{u}}_{{{\varvec{IF}}}} - e_{00} } \right)}}$$

The three error terms are determined by a calibration process based on the solution of a system of three equations with three unknowns. The three equations are established by Eq. (6) and terminating the analog front-end with an open circuit, a short circuit and a load of 50 Ohms whose reflection coefficients ${\Gamma }_{{{\text{open}}}}$, ${\Gamma }_{{{\text{short}}}}$ and ${\Gamma }_{{{\text{load}}}}$ are known and whose analytical signals ${\varvec{u}}_{{{\varvec{IF}}\_{\varvec{open}}}}$, ${\varvec{u}}_{{{\varvec{IF}}\_{\varvec{short}}}}$ and ${\varvec{u}}_{{{\varvec{IF}}\_{\varvec{load}}}}$ are measured. It is important to mention that to solve the system of equations the analytical signal ${\varvec{u}}_{{{\varvec{IF}}}}$ obtained through the Hilbert transform of the measured real signal ($u_{IF}$) is used. To which before the application of the transform is added a Hamming window to minimize the edge detection effects caused by the Hilbert transform. That are visually observed as a peak in the signal obtained from the transform at the place where it is has a discontinuity in the signal to which the transform was applied^39^. Because of this, for the generation of the measured real signal a chirp signal of bandwidth greater than the bandwidth of interest is used to avoid the presence of edge detection effects within the bandwidth of interest, since these effects are presented in the ends of the analytical signal.

### ADC module and processing unit

The analog front-end is designed to generate an IF signal with a frequency in the order of Hz, so the ADC can work with a low sampling frequency. In addition, the ADC module includes a conditioning stage designed to add DC offset and amplify the IF signal. The DC offset allows the signal to have only positive values while the amplification helps the signal to cover the entire input voltage range of the ADC. The ADC used for this work is a unipolar ADC integrated into the Arduino UNO microcontroller.

The processing unit is implemented in the same Arduino, so that it is possible to take better advantage of Arduino’s resources since in addition to an ADC, Arduino has a TTL-USB converter that allows direct communication with a PC through an RS-232 interface.

### ZigBee network and sensor connection to network

For this work a ZigBee network was implemented whose diagram is shown in Fig. 1a. The network function is to allow remote control of the sensor node through a client PC. The network allows the exchange of digital data between the client PC and the sensor node. The network consists mainly of a Coordinator module and an End device module. The Coordinator module connects to the client PC because it is responsible for communicating commands to the network while the End device module connects to the sensor node because it is responsible for acting as transceiver of the sensor node. The modules feature an RS-232 interface that allows them to connect with a digital device. In order to connect the Coordinator to the client PC and the End device to the sensor node, a converter or adapter is necessary to use the digital interface.

### Data processing

As mentioned above, the sensor node is integrated into a WSN that allows remote measurements of the $\Gamma$ generated at the input of a microwave sensor. For this, both the sensor node and the client PC individually must carry out a specific process so that they can work in a synchronized manner.

For this work, it is desired to obtain the Γ generated by the sensor in a bandwidth ranging from 2.4 to 3.4 GHz. However, the synthesizer is programmed to generate chirp signals with a bandwidth ranging from 2.3 to 3.5 GHz because, as mentioned above, bandwidth greater than the bandwidth of interest should be used to mitigate from the measurement the effects of edge detection generated by the Hilbert transform. It was identified that good results are achieved if the measurement bandwidth is increased by 100 MHz at each end with respect to the bandwidth of interest. Because of this and due to the sampling frequency achieved by the sensor node connected to the network, the $u_{IF} \left( n \right)$ signal will consist of 1183 samples. Therefore, to ensure that the 1183 samples corresponding to the $u_{IF} \left( n \right)$ signal were obtained in each measurement, it was determined to collect 2000 samples from the sensor node and manually select the 1183 samples desired.

**Figure 3:**
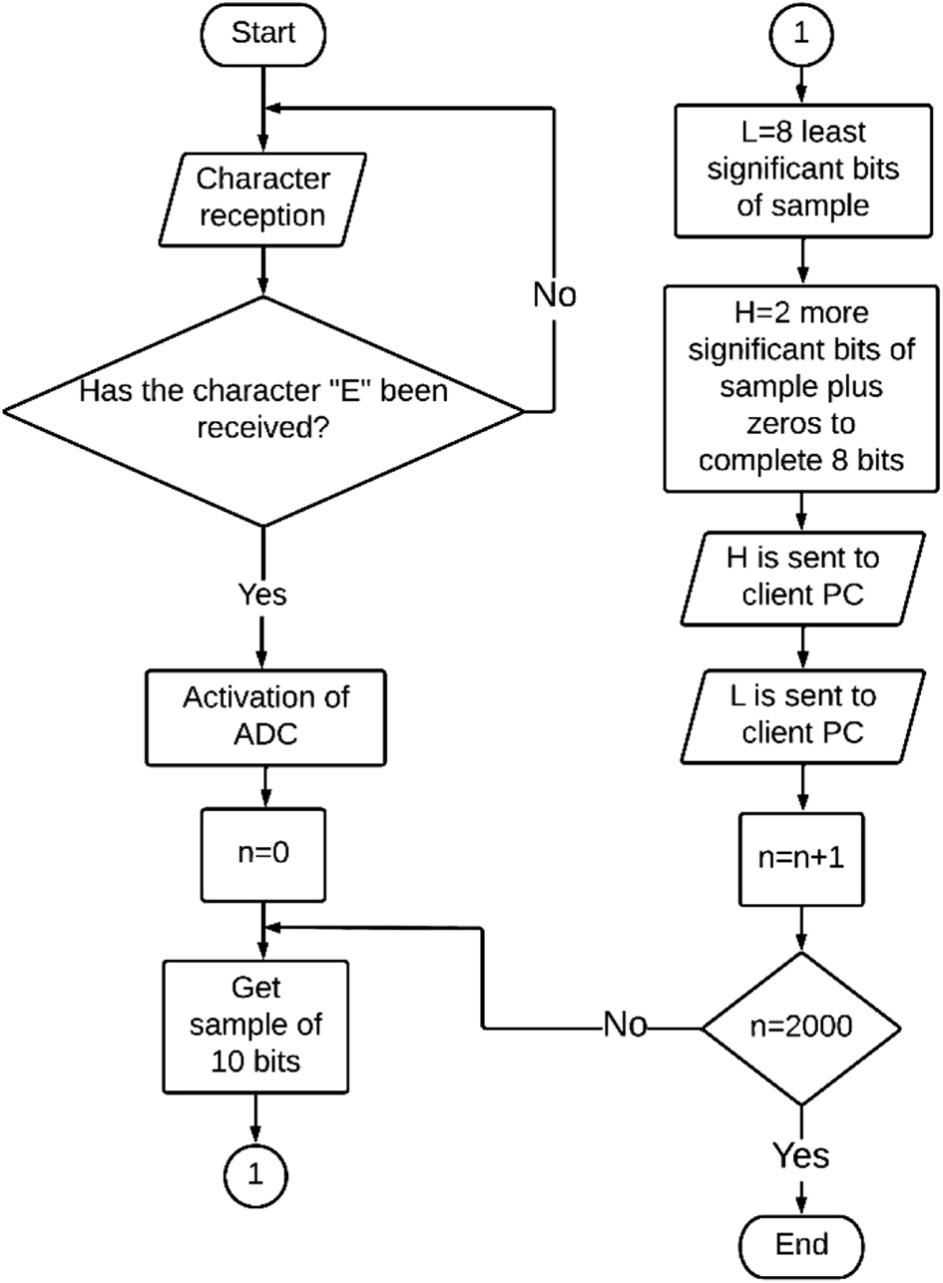
Process flow diagram made by the sensor node.

The process carried out by the sensor node is shown in a flow diagram in Fig. 3. The flowchart shows that at the beginning the sensor node is always ready to receive characters sent from the client PC. Once the start character "E" is received, the processing unit of the sensor node allows the ADC to begin the process of sampling the $u_{IF} \left( t \right)$ signal at the same time as initializing the variable n (counter of sent samples). Because the ADC generates 10-bit samples, once a sample is generated it is divided into two bytes to be able to send the sample through the ZigBee network, since the ZigBee network transmits bytes. In such a way that the data L and data H are generated, where the data L is composed of the 8 least significant bits of the sample and the data H is composed of the 2 most significant bits of the sample and zeros to complete the 8 bits. Once the data H and L are ready, they are sent to the client PC by first sending the data H and then the data L. Once data H and data L are sent, the variable n is increased by 1 and revised if n = 2000, since, if the statement is true, it will be understood that 2000 samples of the $u_{IF} \left( n \right)$ signal have already been sent to the client PC and the process of the sensor node will be completed. Otherwise, a sample of the ADC will be taken again and the previous process will be performed again.

**Figure 4:**
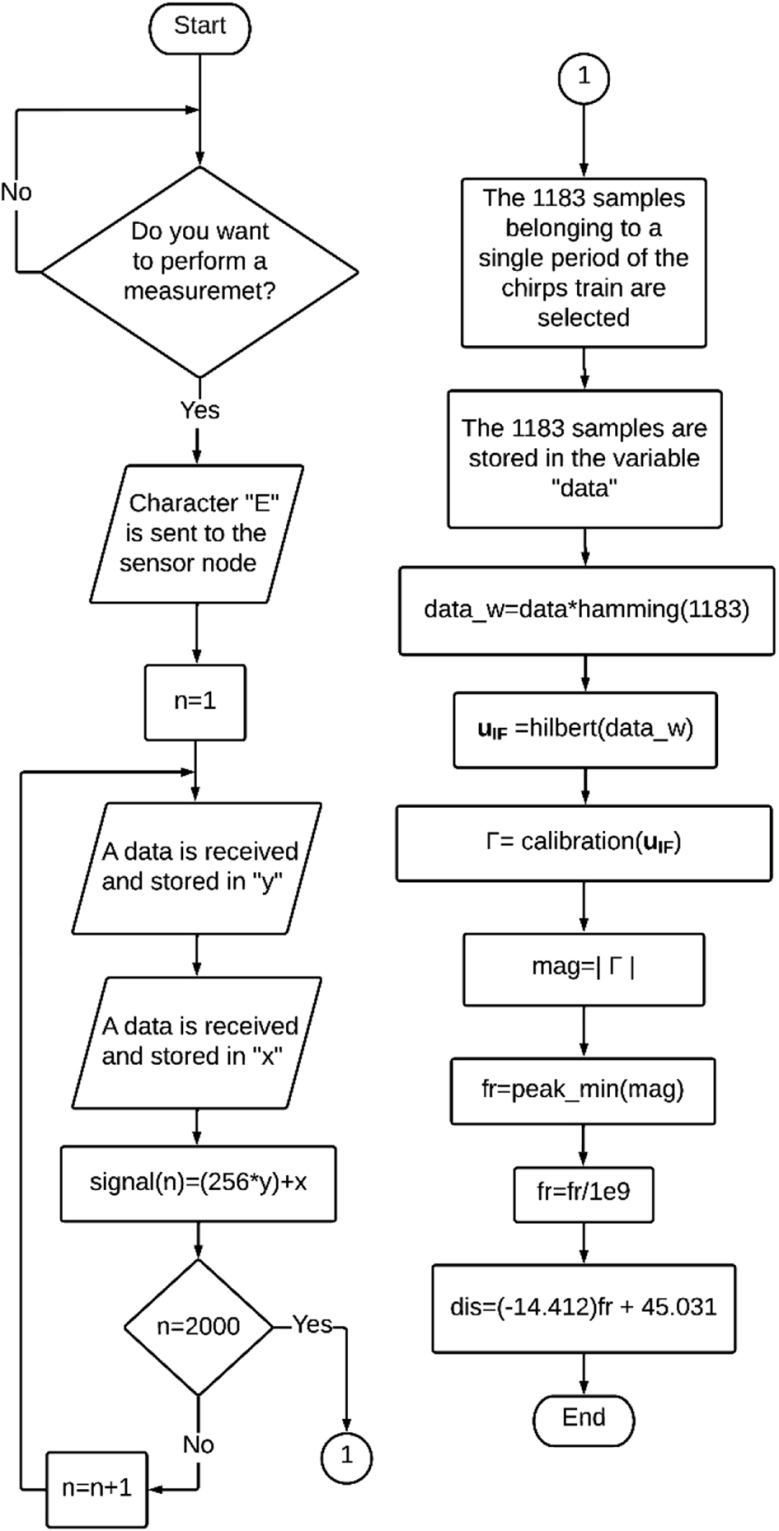
Process flow diagram executed by the client PC.

On the other hand, Fig. 4 shows by means of a flow diagram the process that the client PC executes to interpret the measurement of the sensor. The flow diagram shows that the client PC process starts by asking the user Do you want to perform a measurement? Once the user decides to perform a measurement, the client PC sends the start character "E" to the sensor node so that it starts with the above process. At the same time the variable n (sample counter) starts at 1. Subsequently, the client PC waits for data H and data L that are sent by the sensor node so that once received the data H is stored in the variable "y" and once received the data L is stored in the variable "x". Having data H and data L stored in the variables "y" and "x", they are used to calculate the value of the array "signal" at position n using the operation presented in the flowchart, which is the value corresponding to the sample obtained on the sensor node and encoded in data H and data L. Then it is checked if n = 2000, in the event that the statement is false, the value of n is increased by 1 and the arrival of the next pair of data H and data L will be waited again, and the previous process will be repeated. On the other hand, if the statement is true, the number of samples needed to calculate Γ will be available so that the next stage of the client PC process can be carried out.

Once the 2000 samples have been obtained, the next step is to manually select the 1183 samples corresponding to the signal $u_{IF} \left( n \right)$. This is done by plotting the 2000 samples stored in the "signal" array and manually locating the points at which a discontinuity occurs. Once two points of discontinuity are located, all the points between the discontinuities are stored in the "data" array, since they are the 1183 desired samples. The next step is to apply a window to the data stored in "data", which for this point can be considered as a signal, using a Hamming window to then store the result in the "data_w" array. Once the "data_w" signal is obtained, the Hilbert transform is applied to obtain the ${\varvec{u}}_{{{\varvec{IF}}}}$ signal, which is a complex signal as explained above. After obtaining the ${\varvec{u}}_{{{\varvec{IF}}}}$ signal, the calibration process is carried out to obtain $\Gamma$, which requires the calculation of the error terms corresponding to the front-end circuit. After calibration, the magnitude of $\Gamma$ is calculated to determine the resonance frequency of the sensor by locating the frequency at which the minimum value of the magnitude of $\Gamma$ is presented within the bandwidth of interest (peak_min). Once the resonance frequency of the sensor is located, it can be used to determine the displacement measured by the sensor using the equation of straight line presented in the flow diagram.

## Results

### Implementation of the proposed system

The sensor was designed to obtain an initial resonance frequency of 3.1 GHz on a substrate (FR4) with a relative permittivity of 4.4, loss tangent of 0.02, dielectric thickness of 1.6 mm and copper of 1 Oz. Sensor dimensions are $$L_{a} = 20$$ mm, $$W_{a} = W_{p} = 26$$ mm, $$W_{s} = 0.8$$ mm, $$L_{{{\raise0.7ex\hbox{$\lambda $} \!\mathord{\left/ {\vphantom {\lambda 4}}\right.\kern-0pt} \!\lower0.7ex\hbox{$4$}}}} = 12$$ mm, $$L_{p} = 15$$ mm, $$Lf = 5$$ mm, $$Wf = 3$$ mm and $$L_{o} = 13$$ mm. The dimensions of the antenna were calculated using the equations present in Balanis^36^.

To verify the correct functioning of the sensor, the simulation of the sensor was made in the full wave simulator HFSS. Figure 6 shows the curve fitting obtained from the resonance frequency graph vs $\Delta L$. The curve fitting is given by the following equation:

7 $$f_{0,HFSS} = \left( { - 0.108*\Delta L} \right) + 3.081$$

The patch was printed on a rectangle of FR4 with dimensions $W_{p} = W_{a} = 26$ mm and $L_{p2} = 33$ mm, when $L_{p2}$ is the length of the substrate that supports the mobile patch and allows the patch to be fixed to a structure.

The patch is fixed to a PLA block as shown in Fig. 2c. The antenna was printed with standard photolithography and covered with a PLA block as shown in Fig. 2c. All PLA blocks allow a sensor with $L_{o} = 13$ mm. The PLA blocks were printed with a triangular pattern and a 10% filling factor on a Flashforge 3D printer. The filling factor is 10% to ensure that the block has a low permittivity value and does not affect the sensor’s electrical response. The PLA blocks allow the sensor to be electrically isolated from the structures that form the crack, as well as reducing measurement errors due to unwanted displacements between the mobile patch and the antenna patch.

Having the sensor ready to be installed in a structure, a setup was manufactured to emulate a crack. Figure 6 shows an artificial crack of variable opening that was used in this work. The artificial crack is composed of two separate tile pieces emulating the crack opening. The tile pieces are movable to obtain crack openings of different dimensions which are measured with a micrometer. For the experiment, a piece of plastic was attached to the side of the sensor housing to push the moving part of the sensor through the micrometer spindle. Subsequently, the micrometer was adjusted so that $\Delta L = 0$ mm.

The analog front-end was implemented by two Wilkinson 3 dB Mini-Circuits ZFSC-2-10G power splitters operating from 2 to 10 GHz, an SFI 2040 isolator operating in a range of 2 to 4 GHz and a Mini Circuits ZEM-4300 mixer that accepts RF signals between 300 MHz and 4.3 GHz and that can generate IF signals ranging from DC to 1 GHz.

**Figure 5:**
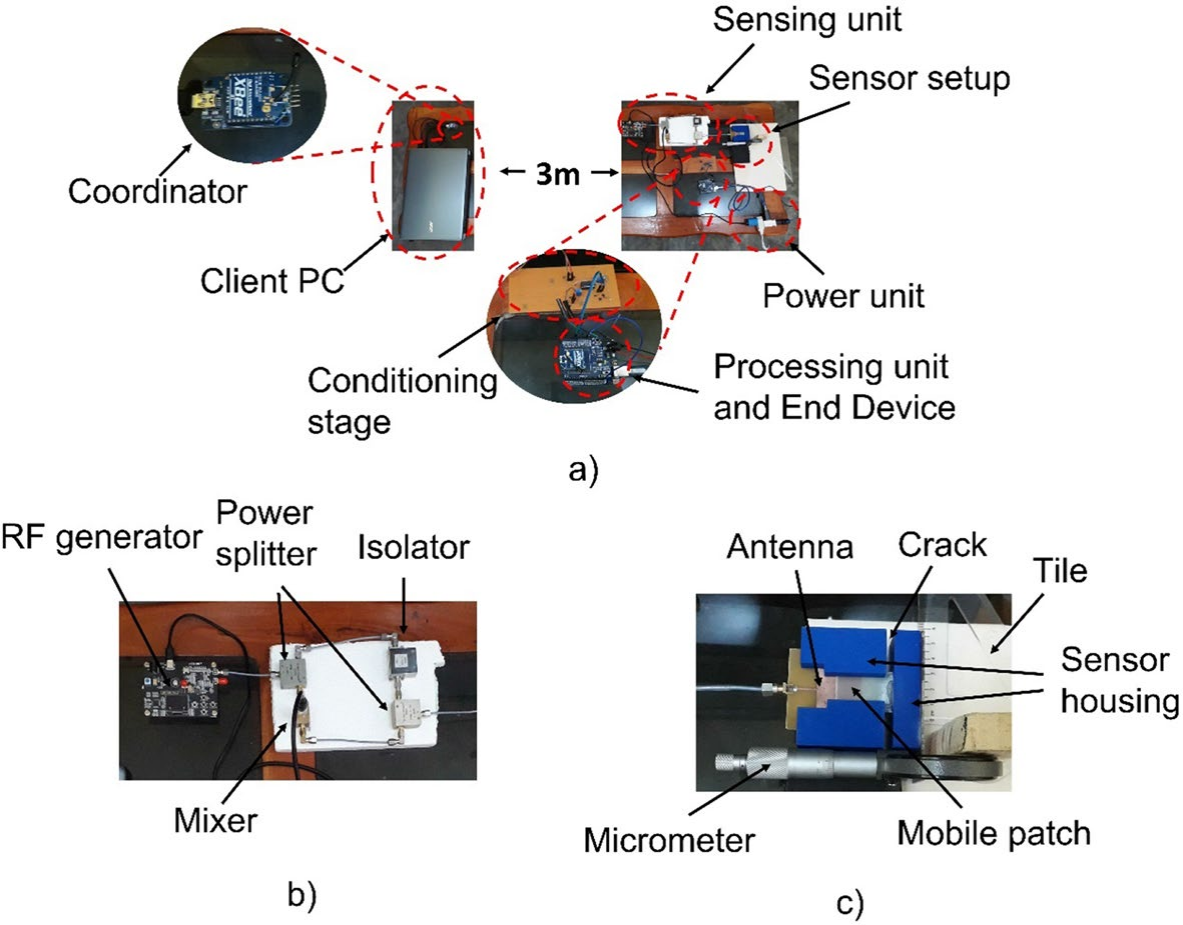
(**a**) Setup of the sensor node integrated in a ZigBee network, (**b**) Sensing unit and (**c**) Setup of the sensor implemented to emulate sensor installed in a cracked structure.

The front-end circuit is powered by a generator as seen in Fig. 5. The RF generator consists of an ADF4351 frequency synthesizer controlled by an STM32F103C8T6 microcontroller via a three-wire SPI interface. The synthesizer has a VCO that can generate output signals with a fundamental frequency between 2200 and 4400 MHz. In addition, frequency dividers are added to generate signals with a frequency range between 35 MHz and 4.4 GHz^40^. The synthesizer allows to generate a chirp signal where the frequency step (minimum step of 10 kHz in generator) and the time step (minimum step of 1 ms in generator) can be varied. Likewise, the synthesizer can vary the output signal power in a range ranging from -4 dBm to 5 dBm. To measure the reflection coefficient, the generator was programmed to generate a chirp signal train of $f_{L} = 2.3$ GHz, $f_{H} = 3.5$ GHz, a period $T = 11$ s and a power -1 dBm.

On the other hand, the output of the front-end circuit, which is located on the IF port of the mixer, is connected to a conditioning circuit made with an LM324 operational amplifier with a non-inverting summing amplifier configuration that allows the IF signal to be adapted to the input voltage range of the ADC.

Figure 5 shows the processing unit that was implemented in Arduino. The processing unit executes a process that allows sending to the client PC 10-bit samples that are obtained from the ADC through two bytes (H and L). The time it takes the two bytes to travel from the sensor node to the client PC across the network is the time that determines the system sample rate. That is, the transmission speed of the RS-232 protocol in conjunction with the transmission speed of the ZigBee protocol, which for this case were both set to 9600 bits/s, are directly those that determine the sampling rate that for this work was $Fs = 107.5$ Hz with which 1183 samples are needed to sample the signal $u_{IF} \left( t \right)$.

In the same Arduino where the processing unit is located, an XBee Shield card is connected to add a ZigBee transceiver (XBee module) to the sensor node, as the XBee Shield adapts the Arduino voltage to the voltage required to power the XBee module, which allows to establish a correct communication between Arduino and the transceiver.

### Experimental test of the system with an artificial crack

**Figure 6:**
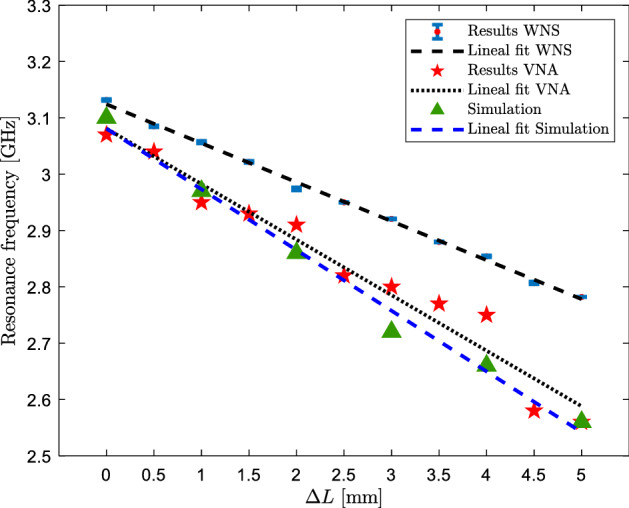
Sensor resonance frequency curve vs $\Delta L$ of simulation, measurement with VNA and measurement with WSN.

To verify the correct functioning of the sensor, the measurement of the sensor was carried out with the VNA-SPARQ 3002E before testing the functionality via WSN. Figure 6 shows the curve fitting representing the resonance frequency measured with the VNA vs the displacement of the sensor incorporated in the set-up in Fig. 5c. The curve fitting is a straight line given by the following equation:

8 $$f_{0,VNA} = \left( { - 0.096*\Delta L} \right) + 3.0809$$

After the sensor was measured with the VNA, the sensor was measured with the sensor node integrated into the WSN. The sensor node was read with the client PC placed 3 m away. In total, 11 openings of the synthetic crack were generated. Each aperture is separated by 0.5 mm from the previous one. For each aperture, 10 measurements of the sensor reflection coefficient were made. The resonance frequency of the sensor was determined for each of the 10 measurements. Figure 6 shows the average measured resonance frequency curve vs $\Delta L$ and the standard deviation represented by an error bar that in all cases it is small enough to ensure the distinction between one case and another; The adjustment curve, which represents the average measured resonance frequency curve vs $\Delta L$ is given as follows:

9 $$f_{0,WSN} = \left( { - 0.069*\Delta L} \right) + 3.124$$

Table 1 shows the average resonance frequency and the calculated standard deviation for each aperture measured with the sensor; the results show that the sensor node allows accurate measurements of the resonance frequency. Since when analyzing the standard deviation, which is a statistical measure indicating that so dispersed is a group of data with respect to its mean, it was obtained that in the best of cases (5 mm) the standard deviation was 4.774e-4 GHz while in the worst case (2 mm) it was 2.834e-3 GHz.

**Table 1:** Average resonance frequency and standard deviation calculated with WSN measurements.

$\Delta L$(mm)	Average resonance frequency (GHz)	Standard deviation (GHz)
0	3.132	1.789e−3
0.5	3.085	1.815e−3
1	3.057	2.348e−3
1.5	3.022	2.081e−3
2	2.974	2.834e−3
2.5	2.950	7.988e−4
3	2.921	9.306e−4
3.5	2.880	1.089e−3
4	2.854	1.640e−3
4.5	2.807	2.492e−3
5	2.782	4.774e−4

Equations (7), (8) and (9) shows that the sensitivity of the simulated sensor, measured with VNA and tested via WSN is 0.108 GHz/mm, 0.098 GHz/mm, and 0.070 GHz/mm respectively. The correlation coefficient (R) and the root mean square error (RMSE) are 0.989 and 0.019 for simulations, 0.958 and 0.032 for measurements with the VNA and 0.998 and 0.005 for the sensor tested via the WSN. From these results it is obtained that the results of the proposed sensor exhibit R close to 1 which indicates that the data are well represented by a line with a low RMSE value.

Table 2 includes the comparison of the results of our work with the experimental results obtained in Xue^31^, Horestani^32^, Rezaee^33^, Soltan^34^and Wang^35^, where a VNA was used as a measuring instrument. When comparing the sensitivity it can be said that the sensitivity shown with the data obtained with the WSN is high since in three of the five papers presented a lower sensitivity is reported. Finally, when comparing R we can say that the measurements of our sensor obtained with the WSN present the value of R closest to 1 which indicates that these data present the most linear behavior which justifies the low RMSE calculated for this case. This indicates that the equation of straight line obtained from its adjustment will be the one that will yield the most accurate estimates of $\Delta L$ as a function of the resonance frequency.

**Table 2:** Comparison of sensor sensitivity, $f_{0}$, R and RMSE between simulation, measurements with VNA, measurements with WSN and works present in the literature.

Serial	Sensitivity (GHz/mm)	$f_{0}$(GHz)	R/RMSE	VNA required	Connecting to a sensor network (network type)
Xue^31^	0.195	2.326	–	Yes	No
Horestani^32^	0.080	1.600	–	Yes	No
Rezaee^33^	0.041	2.800	–	Yes	No
Soltan^34^	0.025	2.400	–	Yes	No
Wang^35^	0.031	0.378	–	Yes	Yes (wire network)
This work	0.098	3.081	0.958/0.032	Yes	No
This work (WSN)	0.070	3.124	0.998/0.005	No	Yes (wireless network)

## Conclusions

In this work, a sensor node integrated into a short-range WSN based on ZigBee technology was presented. The sensor node measures the opening of structural cracks through the reflection coefficient measured at the input of a microwave sensor from a port which is placed between the crack. The sensor node uses a displacement sensor based on a patch antenna to measure the crack opening and an analog front-end circuit to perform the sensor reading. The analog front-end consists of a synthesizer, an isolator, two Wilkinson-type power dividers and a mixer. The front-end is powered by a generator programmed to generate a chirp signal train of $f_{L} = 2.3$ GHz, $f_{H} = 3.5$ GHz, a period $T = 11$ s and a power -1 dBm. The output signal of the front-end circuit is digitized with the ADC contained in an Arduino UNO. The system includes a processing unit implemented in Arduino and a transceiver based on an XBee module. On the other hand, the network consists of a client PC made through a PC with Matlab 2018 connected to the sensor node through a Coordinator device implemented by an XBee module and a TTL-USB converter.

## Data Availability

The datasets used and/or analyzed during the current study available from the corresponding author on reasonable request.
